# CD4/CD8–p56^lck^ Induced T-Cell Receptor Signaling and Its Implications for Immunotherapy

**DOI:** 10.3390/biom15081096

**Published:** 2025-07-29

**Authors:** Andres Oroya, Christopher E. Rudd

**Affiliations:** 1 Département de Médicine, Université de Montréal, Montréal, QC H3C 3J7, Canada; carlos.andres.oroya.lazo@umontreal.ca; 2 Département de Microbiologie, Infectiologie et Immunologie, Université de Montréal, Montréal, QC H3T 1J4, Canada; 3 L’unité, Signalisation Cellulaire en Immunothérapie, Centre de Recherche de l’Hôpital Maisonneuve-Rosemont (CR-HMR), Montréal, QC H1T 2M4, Canada; 4 Institut Universitaire d’Hématologie-Oncologie & Thérapie Cellulaire de Montréal, Hôpital Maisonneuve-Rosemont, Montréal, QC H1T 2M4, Canada; 5 Department of Medicine, McGill University, Montréal, QC H3A 2B4, Canada; 6 ImmunAb Research Inc., 2300 Blvd Alfred-Nobel, Montréal, QC H4S 2A4, Canada

**Keywords:** T-cell receptor, signal transduction, protein phosphorylation, p56^lck^, CAR T-cell, cancer immunotherapy

## Abstract

T-cells constitute an essential component of the adaptive immune response, mount a protective response against foreign pathogens and are important regulators of anti-tumor immunotherapy. In this context, the activation of T-cells and chimeric antigen receptor (CAR)-expressing T-cells is orchestrated by various signaling pathways, involving the initiation of a protein tyrosine phosphorylation cascade. For T-cells, this involves initiation of the phosphorylation cascade via *src*-related protein-tyrosine kinase p56^lck^, which we show to associate with the co-receptors CD4 and CD8 for the induction of a phosphorylation cascade needed for the activation of T-cells. Likewise, p56^lck^ phosphorylation of the antigen receptor immunoreceptor tyrosine-based activation motifs (ITAMs) and key CD28 tyrosine motifs ensures the functionality and the survival of CARs, while their phospho-targets are also inhibited by PD-1, a key component of the immune checkpoint blockade. This review covers historic and current elements of our knowledge of CD4/CD8–p56^lck^-induced activation events and their importance to the development of CAR T-cell immunotherapies.

## 1. Introduction

T-cells constitute a vital component of the immune system, mediating the adaptive immune response and playing a significant role in infections and cancer [1,2]. Proper T-cell activation leads to the initiation of different cell functions, such as proliferation, differentiation into effector and memory cells, migration, and regulation of other cells [3]. Therefore, it is essential to understand the receptors and intracellular mechanisms that regulate T-cell activation. This latter process is controlled by multiple signals, where the T-cell receptor signaling pathway is widely recognized as “signal one”, which initiates antigen-specific responses. We first showed that the CD4 and CD8 co-receptors bind to the intracellular protein-tyrosine kinase p56^lck^ complex [4,5,6]. Further, we and others showed that this complex and p56^lck^ can initiate the phosphorylation cascade in T-cells that is needed for activation [5,6,7,8]. This is followed by a mechanistic role of p56^lck^ in the phosphorylation of the co-receptor CD28 for the binding of phosphatidyl inositol 3-kinase and the adaptor GrB-2 [9,10,11] as well as its actual binding to the co-receptor in the mediation of co-stimulation [12]. By contrast, inhibitory co-receptors such as CTLA-4 and PD-1 negatively regulate T-cell activation [9,13,14,15]. TCR signaling is complemented by these additional co-receptor pathways to ensure that the T-cell responses are fine-tuned with enhanced cell survival [14,16,17].

Given the central importance of T-cells in the anti-cancer immune responses, extensive research is being conducted on these signaling pathways in targeted therapeutics against cancer. In this context, the downstream targets of p56^lck^, such as the phospho-sites on immunoreceptor tyrosine-based activation motifs (ITAMs) and CD28, play key roles in ensuring the functionality and survival of chimeric antigen receptors (CARs) [18,19]. Similarly, PD-1 targets components of the CD4/CD8–p56^lck^-initiated phosphorylation cascade in the immune checkpoint blockade [20].

Here, we describe and discuss certain key aspects of CD4/CD8–p56^lck^-initiated phosphorylation in the TCR signaling pathway as well as their implications for the development of CAR T-cell immunotherapy.

## 2. The First Steps in the TCR Signaling Pathway

**Figure 1:**
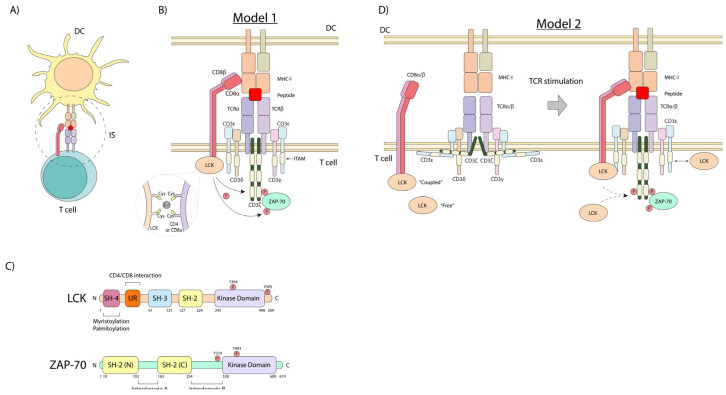
The T-cell-APC complex, the structure of p56^lck^ and ZAP-70, and models of the CD4/CD8–p56^lck^-induced TCR signaling complex. (**A**) DC-mediated activation of T-cells during the antigen presentation process. (**B**) Model 1: Peptide–MHC–TCR interaction allows the recruitment of the CD8 (or CD4) co-receptor that is bound to p56^LCK^ kinase. This interaction is mediated by cysteines and coordinated by a Zn^2+^ ion. This brings LCK into proximity and phosphorylates the ITAMs of the CD3 complex, promoting the recruitment and phospho-activation of ZAP-70 kinase. (**C**) Structure of LCK and ZAP-70 kinases. (**D**) Model 2: pMHC-TCR ligation induces conformational changes in CD3e and CD3z, releasing their cytoplasmic tails, allowing the recruitment of LCK to mediate the tyrosine phosphorylation of CD3z and ZAP-70. DC: dendritic cell, IS: immunological synapse, SH: Src homology domain, UR: unique region.

T-cell activation is initiated by the ligation of the T-cell receptor (i.e., otherwise known as the antigen receptor), which is accomplished by the presentation of an antigen-derived peptide by antigen-presenting cells (APCs), such as dendritic cells (DCs) [21,22]. DCs generate small peptides that are presented by major histocompatibility complex I or II (MHC-I or -II) antigens [23] (Figure 1A). This peptide–MHC (pMHC) complex is recognized by the TCR, involving the co-recruitment of CD8 or CD4 co-receptors, which bind to non-polymorphic, conserved regions of the MHC-I or MHC-II, respectively [24] (Figure 1B).

CD4 and CD8 are co-receptors found on the surface of T-cells that play a crucial role in the adaptive immune response by binding to non-variable regions of the major histocompatibility complex (MHC) antigens. The CD4 co-receptor is found on helper T-cells and binds to class II MHC molecules, which present extracellular pathogen-derived peptides that initiate the adaptive immune response. The CD8 co-receptor is expressed on cytotoxic T-cells and interacts with class I MHC molecules displaying peptides from intracellular pathogens or aberrant self-proteins, enabling the killing of infected or abnormal cells. The binding of the CD4 or CD8 co-receptor to the appropriate MHC class molecule stabilizes the interaction between the T-cell receptor and the MHC–peptide complex, facilitating T-cell activation and an effective immune response [25]. They also expand the T-cell repertoire with activation [26].

As we showed decades ago, the intracellular tails of the CD4 and CD8 co-receptors bind the Src family kinase p56^lck^ protein-tyrosine kinase (LCK, lymphocyte cell-specific protein-tyrosine kinase) [4,5,6,8]. This, as we also proposed, initiates a downstream protein-tyrosine phosphorylation cascade needed for T-cell activation [18]. CD4 and CD8α contain a conserved CxCP motif that is required for p56^lck^ binding [27]. Cysteines from this motif can interact with those in the p56^lck^ CxxC motif located in the unique region through a zinc^2+^ ion [28,29] (Figure 1B,C). Moreover, this interaction is also stabilized by ionic interaction through basic residues in CD4 and CD8α and acid amino acids in p56^lck^, preceding the cysteine-containing motifs [29]. LCK binds CD4 in a zinc-dependent manner [27], and it is debatable whether the cross-linking of CD4 increased the LCK activity [30].

p56^lck^ has an N-terminal region, which is myristoylated and palmitoylated for membrane anchoring, a unique co-receptor binding region, followed by an SH3 and SH2 domain for intra- and extra-binding, and finally, a catalytic kinase (SH1) domain (Figure 1C). Its enzymatic function is controlled positively by the phosphorylation of tyrosine Y-394 in the activation loop of the kinase, and negatively by Y-505 phosphorylation in the C-terminal tail. Y-505 phosphorylation promotes an intramolecular “closed” conformation, primarily via its SH2 domain binding to Y-505. p56^lck^ activity is governed by the activating Y-394 and inhibitory Y-505 phosphorylation, which in turn is mediated via kinases like Csk and phosphatases such as CD45 [18,31,32].

T-cell activation can also induce the phosphorylation of CD4 and its internalization [33,34] (Figure 1B). Notably, CD4 contains a dileucine motif that is masked when interacting with LCK, preventing clathrin-dependent endocytosis of CD4 [27]. Thus, the interaction between LCK and CD4 and the stability of LCK may enhance TCR stimulation by maintaining surface levels of CD4. The intracellular tails of the CD4 and CD8 co-receptors therefore were shown to bind the Src family kinase p56^lck^ (LCK), initiating a downstream protein-tyrosine phosphorylation cascade crucial for T-cell activation.

The identification of the CD4/CD8–p56^lck^ complexes served as a model for how receptors lacking intrinsic catalytic activity could initiate cellular signaling and served as a model for other receptor src–kinase interactions [18]. For example, the B-cell receptor (BCR) and Fc receptors recruit src family members such as Lyn to generate activation signals. Similarly, growth factor receptors like EGFR, PDGFR, and VEGFR interact with Src, Yes and Fyn in their signaling pathways. It also placed an emphasis on the role played by p56^lck^ in the function of other T-cell receptors. In this context, p56^lck^ can also associate with CD28 in a manner that bridges it with protein kinase C-θ (PKC-θ) [12]. PKC-θ serves as a control marker for the central supra-molecular activation complex (SMAC) that forms during the contact of T-cells with dendritic cells [35,36]. In our original model, we proposed that during antigen presentation, the recruitment of CD4 and CD8 by binding to non-polymorphic regions of MHC antigens would bring p56^lck^ into proximity with the interaction contact site, leading to the phosphorylation of the antigen receptor complex [4,5,6,18] (Figure 1B). Consistent with this model, subsequent studies have shown that while the TCR signaling has limited effects on the activity status of p56^lck^, the constitutively active kinase is needed and responsible for TCR signaling [37].

However, it is also noteworthy that p56^lck^ may also promote receptor-interaction events within T-cells (Figure 1D). Using total internal reflection/Förster resonance energy transfer microscopy, a two-stage interaction between the TCR, CD8, and MHC–peptide complex has been observed [38]. The early interaction between the CD3ζ chain and CD8 occurred independently of CD8 binding to the MHC. Further and importantly, this early interaction required CD8 to be associated with the protein kinase p56^lck^. However, “free” LCK has been reported to have higher levels of catalytic activity and may play an important early role in the activation process [38,39]. Overall, the functional relevance of the receptor-bound and free forms remains under investigation. The one question is whether the “free” p56^lck^ is truly unbound to a receptor or whether it might also interact in some manner with another unidentified receptor or binding partner such as CD28.

Unlike CD4, the CD8 co-receptor is a heterodimer comprising two subunits, CD8α and CD8β. The CD8α subunit binds LCK in a zinc-dependent manner [27,29] (Figure 1B), while the CD8β cytoplasmic tail may act to improve CD8α-bound p56^lck^ binding for optimal activity [40,41] (Figure 1B). Interestingly, the CD8α chain can also form homodimers, which may negatively affect TCR stimulation [42,43]. Further, one study demonstrated that CD8αα homodimers and CD8αβ heterodimers showed no differences in their binding to MHC-I [44], while another study found the extracellular domain of CD8β to be important for the CD8–MHC-I interaction in thymocytes [41]. The exact role and the spectrum of the differential expression of CD8αα homodimers versus CD8αβ heterodimers remain unclear.

As a complementary mechanism, it has been proposed that the cytoplasmic tails of the CD3ζ and CD3ε chains are membrane-embedded in the inner face of the plasma membrane [45,46] (Figure 1D). This interaction occurs through the binding of basic amino acids in their cytoplasmic tails to negatively charged lipids, such as phosphatidylserine (PS) and phosphatidylinositol species cytoplasmic tail from plasma membrane [47] (Figure 1D). The TCR-pMHC signaling promotes the release of this tail, leading to the exposure of sites for p56^lck^-induced phosphorylation [45]. Overall, this mechanism provides a way for the various receptor cytoplasmic regions to become accessible to p56^lck^ for phosphorylation.

## 3. Microdomains

Possibly related to this, p56^lck^ and CD8/CD4 have been reported to localize in glycosphingolipid-enriched microdomains (GEMs), or lipid rafts. These domains are specialized membrane regions that are rich in cholesterol, glycosphingolipids and sphingomyelin [48]. Although their roles in the TCR signaling pathway remain unclear, they are enriched with src-related kinases like p56^lck.^ Further, it has been reported that TCR and CD4/CD8 colocalize in rafts upon TCR engagement [33]. The functional significance of this localization is debated. Some studies suggest that raft association facilitates p56^LCK^ activation and subsequent TCR phosphorylation [33]. Further evidence supporting the importance of raft association comes from the observation that a raft-excluded p56^LCK^ mutant failed to phosphorylate the TCR [35]. LAT/Grb2/Sos1 mediators can undergo condensation involving coupling between the lipid and protein forms of phase separation [49]. The uncoupling of lipid domains from these protein condensates abrogates T-cell activation.

However, others have proposed that the inhibitory CBP–PAG–CSK complex within rafts may limit kinase activity [34]. Considering that in resting cells, TCR is not strongly raft-associated [34], it is likely that these microdomains play a role in sustaining, rather than initiating, TCR signaling. In this context, we and others have shown that CD28 and the activation-induced inhibitory co-receptor CTLA-4 profoundly altered the surface expression of membrane rafts during T-cell activation [50]. CD28 increased the presence of the linker of activated T-cells (LAT) in purified membrane rafts, while CTLA-4 co-ligation effectively blocked this increase. Further, the reversal of the CTLA-4 block with CD3/CD28 ligation was accompanied by an increase in surface raft expression and the associated LAT. Similarly, the Bluestone lab showed that the co-ligation of CTLA-4 with the TCR decreased the level of TCRζ chain in rafts [51]. While the roles of GEMs in TCR signaling remain a subject of debate, accumulating evidence suggests these membrane microdomains facilitate co-localization and interactions among key signaling molecules like p56^lck^, CD4/CD8, TCR, CD28, CTLA-4 and LAT, potentially sustaining TCR signaling events.

## 4. T-Cell Subsets

As T-cells mature, it has been reported that the stoichiometry of the p56^lck^ interaction increases with CD8 but not with CD4 [52]. This is also in keeping with reported differences in activities associated with the two co-receptors [5,52]. Further, this increase in binding correlates with greater self-reactivity in peripheral CD8+ T-cells when compared to CD4+ T-cells [52]. When bound to CD8, p56^lck^ has been reported to be dispensable for the antiviral and anti-tumor activities of cytotoxic T-cells in mice. Instead, it operates to facilitate CD8+ T-cell responses to suboptimal antigens. In contrast, LCK bound to CD4 is essential for efficient development and function of helper T-cells via a kinase-independent stabilization of surface CD4. In this context, the binding of p56^lck^ has been shown to impair CD4 endocytosis [53,54]. In this manner, the interactions between the co-receptors and LCK emerge as promising targets for immunomodulation [55]. Overall, the distinct levels of p56^lck^ binding to their co-receptors in CD4+ and CD8+ T-cells suggest that targeted modulation of these interactions could selectively enhance or suppress specific T-cell responses. These findings underscore the importance of a detailed understanding of the nuanced interactions with co-receptors in orchestrating cell-subset-specific functions and potentially in treating immune-related disorders.

## 5. Steps in p56^lck^-Initiated TCR Downstream Signaling

**Figure 2:**
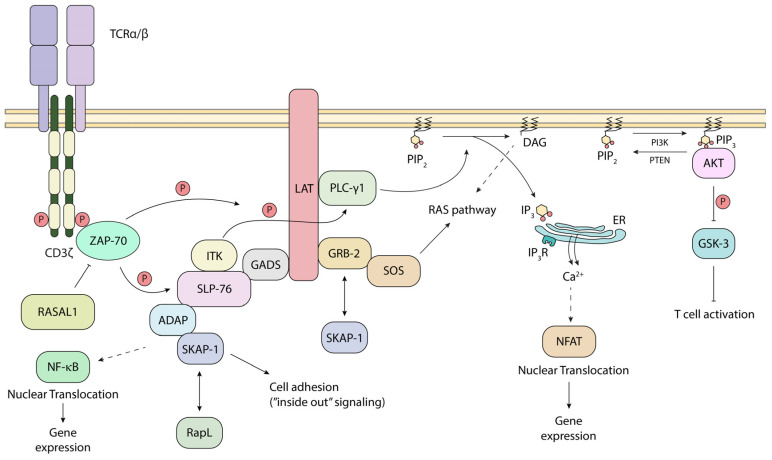
CD4/CD8–p56^lck^-initiated downstream events. Upon phospho-activation of ZAP-70, it phosphorylates LAT, allowing the recruitment of PLC-g1, GRB-2 and GADS branching in the TCR SP. ZAP-70 activity can be limited by RASAL1. TCR triggering modulates the RAS pathway, intracellular Ca2+ signaling, cell adhesion through ADAP-SKAP-1 and the AKT-GSK-3 axis. PIP2: phosphatidylinositol-4, 5-bisphosphate, PIP3: phosphatidylinositol-3, 4, 5-triphosphate, DAG: diacylglycerol, ER: endoplasmic reticulum, IP3R: IP3 receptor.

In now classical studies, p56^lck^ was shown by ourselves and others to phosphorylate the chains of the TCR, and specifically to phosphorylate the ITAMs that serve as docking sites for the 70 kDa zeta-chain-associated protein kinase (ZAP-70) [5,56,57,58] (Figure 2). The CD3ζ chains each possess 3 ITAMs, while the other CD3 subunits each have a single ITAM, resulting in a total of 10 ITAMs per TCR complex [56,59]. The ITAM consensus sequence is (D/E)xxYxx(I/L)x6–8Yxx(I/L) (Figure 1B). ZAP-70 has two tandem SH2 domains that bind to the tandem tryosines within the ITAM motif. Further, once recruited, ZAP-70 is subsequently phospho-activated by p56^lck^ on its tyrosine 493 [60] (Figure 1C). Consistent with its importance, mutation of the Y-493 site impairs the ability of ZAP-70 to be activated by p56^lck^. This recruitment and activation of ZAP-70 is the second step in TCR signaling. Additionally, our laboratory has recently identified RASAL1, a GTPase-activating protein, as a novel negative regulator of ZAP-70 (Figure 2). It binds the kinase domain of ZAP-70 upon TCR stimulation, diminishing ZAP-70 activity [61].

p56^lck^-initiated ZAP-70 recruitment to the TCR is also accompanied by the phosphorylation of immune cell adaptors such as LAT and SLP-76 [32,62,63]. In this way, ZAP-70 is considered more effective and specialized than p56^lck^ in the phosphorylation of key motifs in these adaptors [52]. LAT, in turn, has multiple tyrosine-based binding sites to recruit other adaptors and enzymes, such as phospholipase C, gamma 1 (PLCγ1), growth factor receptor-bound protein 2 (GRB-2), and grb2-related adaptor downstream of Shc (GADS) [32] (Figure 2). GAD binds with high affinity to the Src homology 2 (SH2) domain-containing leukocyte protein of 76 kDa (SLP-76; also known as LCP2, lymphocyte cytosolic protein) [64]. SLP-76 also binds to Tec kinases, resting lymphocyte kinase and inducible tyrosine kinase [65]. Together, they mediate the phosphorylation and activation of PLCγ1. PLCγ1, in turn, acts to hydrolyze PIP2 into IP3 and DAG. IP3 then binds the IP3 receptors on the membrane of the endoplasmic reticulum (ER), triggering the intracellular phase of calcium release into the cytoplasm [66,67]. A LAT-deficient T-cell line showed defects in the tyrosine phosphorylation of PLCγ-1 and calcium mobilization [42]. Further, we showed that the SLP-76 sterile α motif (SAM) and individual H5 α helix further mediates oligomer formation for micro-clusters and T-cell activation [68]. These signaling events ultimately result in the translocation of the nuclear factor of activated T-cells (NFAT) into the nucleus, leading to IL-2 expression [69,70]. This intricate network, initiated by p56^lck^, ensures that T-cells mount a robust response against pathogens and cancer neoantigens.

Further downstream, SLP-76 binds to another immune ADAP, which then binds to another immune adaptor termed SKAP-1 [71,72]. ADAP interacts with SKAP1 primarily via SKAP-1 SH3 domain binding to ADAP proline residues, while the ADAP SH3 domain also binds to a noncanonical motif in SKAP1 [73,74]. We showed that SKAP1 interacts with and modulates the activity of polo-like kinase 1 (PLK1), a crucial regulator of mitosis and cell cycle progression in mammalian cells [75]. During mitosis, PLK1 activates the phosphatase Cdc25C, which is a positive regulator of the Cdc2–cyclin B1 complex. The Cdc2–cyclin B1 complex is a cyclin-dependent kinase that governs the G2/M phase transition of the cell cycle. Additionally, PLK1 contributes to the exit from mitosis by regulating the anaphase-promoting complex (APC) [76,77,78].

Our experimental data indicated that PLK1 binds to the N-terminal serine residue at position 31 (S31) of SKAP1, and this interaction is necessary for optimal PLK1 kinase activity. We showed that the knockdown of SKAP1 using siRNA resulted in a reduced rate of T-cell division, accompanied by a delay in the expression of PLK1, cyclin A, and phosphorylated histone H3 (pH3), a marker of mitosis. By contrast, the reconstitution of the knockdown cells with wild-type SKAP1, but not the SKAP1 S31 mutant, restored the normal cell division rates. These findings suggest that SKAP1, through its interaction with PLK1 and regulation of PLK1 activity, plays a crucial role in optimal cell cycle progression and mitotic events, which are essential for T-cell clonal expansion in response to antigenic stimulation.

Lastly, we also demonstrated that ADAP and its binding to SLP-76 are coupled to the costimulatory function of the LFA-1 (lymphocyte function-associated antigen-1) integrin in T-cells [79]. We found that ADAP and SLP-76–ADAP binding are linked to LFA-1 co-stimulation of interleukin-2 (IL-2) production, F-actin clustering, cell polarization, and T-cell motility. Our results showed that the enhancement of anti-CD3-induced IL-2 production by LFA-1 was completely dependent on the binding between SLP-76 and ADAP. Furthermore, we discovered that anti-CD3 stimulation required CD11a (a subunit of LFA-1) ligation by either antibody or ICAM1 (intercellular adhesion molecule-1) to cause T-cell polarization. ADAP augmented this polarization induced by anti-CD3/CD11a, but not by anti-CD3 alone. Overall, each of these interactions are needed for adhesion and T-cell proliferation [72,79,80,81,82]. NMR spectroscopy and MST data indicate that the N-terminal SH2 domains within a ZAP-70–tandem-SH2 construct are also interaction sites for phosphorylated ADAP-hSH3 (N) with intermediate binding affinity [83]. Each of these studies points to a complex set of interactions amongst many players in the CD4/CD8–p56^lck^, or some instances, free Lck in the protein-tyrosine phosphorylation activation cascade, leading to the combined phosphorylation of enzymes and adaptors in T-cells.

## 6. CD4/CD8–p56^lck^ and CAR T-Cell Immunotherapy

**Figure 3:**
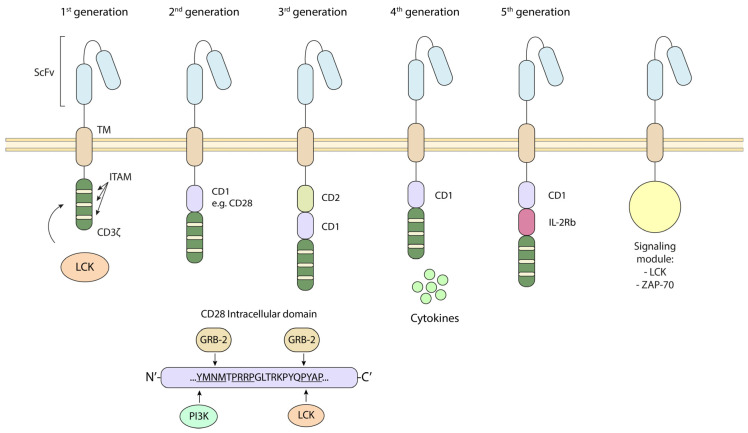
The inclusion of CD4/CD8–p56^lck^-targeted sequences for the successful design of novel CAR T-cells in immunotherapy. Distinct CAR generations contain p56^lck^ targets such as CD3z ITAMs and a CD28 intracellular domains such as the pYMNM motif for binding to PI 3K or the YAP motif that binds LCK. New generations of CARs contain signaling modules consisting of a catalytic portion of LCK or ZAP-70 that can mediate signal transduction. First-generation CARs possess only a CD3ζ motif, while second- and third-generation CARs include one and two co-stimulatory domains, respectively. Each are phosphorylated by p56^lck^. These cytoplasmic tails or domains often from CD28 or 4-1BB. In the case of CD28, we and others have defined key domains that promote CAR T-cell survival [9,11,86,87]. More recently, fourth-generation CAR T-cells, also known as TRUCKs, can also produce desired cytokines such as IL-2, IL-12, IL-15, IL-18, etc., while fifth-generation CARs incorporate other domains that can activate cytokine signaling. ScFv: single-chain variable fragment, TM: transmembrane region, CD: co-stimulatory domain.

The discovery of the CD4/CD8–p56^lck^ complexes and the identification of the targets of the initiated phosphorylation cascade have given rise to the development of chimeric antigen receptor (CAR) T-cell immunotherapy in combating cancer and infections [84,85] (Figure 3). CARs are engineered proteins incorporating intracellular motifs from signaling molecules such as CD3ζ and CD28, alongside regions for antigen recognition (generally a single-chain variable fragment, scFv, generally from antibodies against tumor-associated or -specific antigens) (Figure 3). CARs are classified based on the incorporated motifs in their cytoplasmic portion. First-generation CARs possess only a CD3ζ motif, while second- and third-generation CARs include one and two co-stimulatory domains, respectively. These cytoplasmic tails or domains are often from CD28 or 4-1BB. In the case of CD28, we and others have defined key domains that promote CAR T-cell survival [9,11,86,87]. We and others showed that the YMNM motif (and an analogous motif in CTLA-4) binds to PI 3K, which, in turn, promotes T-cell survival [11,87,88]. Similarly, CD28 binding to p56^lck^ via a distal tyrosine-based motif connects the co-receptor to PKC-θ and also plays a role in promoting cell survival [12] (Figure 3, lower panel). On the other hand, the potency of CD28 co-stimulation has been reported to augment, whereas 4-1BB co-stimulation reduces, exhaustion induced by persistent CAR signaling [89]. More recently, fourth-generation CAR T-cells, also known as TRUCKs, can also produce desired cytokines such as IL-2, IL-12, IL-15, IL-18, etc. Fifth-generation CARs incorporate other domains that can activate cytokine signaling with antigen stimulation, bypassing the need for cytokine stimulation [70]. Moreover, while the CD28 transmembrane portion (TM) is commonly used in CARs, CD8α TM or hinge regions have also proven advantageous in CARs against tumor antigens, and in our lab, against SARS-CoV-2 antigens [90,91]. These chimeric combinations can promote better activation and function for robust anti-tumor responses [92].

In addition to the inclusion of motifs identified as targets of p56^lck^, the kinase and other molecules, such as ZAP-70, LAT, and SLP-76, are essential for CAR activity. Further, certain proximal signaling CARs, such as a ZAP-70 CAR, can activate T-cells and eradicate tumors in vivo while bypassing upstream signaling proteins, including CD3zeta [93]. This combined with a cooperative link to the downstream targets of ZAP-70, namely LAT and SLP-76, has been engineered to produce more effective reagents [93] (Figure 3). Potentially, mutating the inhibitory phosphorylation site Y-505 on p56^lck^ could boost LCK-coupled CAR signaling [16]. p56^lck^ can also operate in the context of CD28-mediated co-stimulation and enhance 4-1BB CAR-CD3 basal phosphorylation. This is associated with increased T-cell levels in blood and improved anti-tumor responses [94].

One key question is the interplay between CARs and endogenous receptors and signaling events. We previously showed that p56^lck^ and p59^fyn^ readily phosphorylate motifs such as the pYMNM motif in CD28 [10,11]. The motif can regulate CD28 binding to phosphatidylinositol 3-kinase (PI 3K), growth factor receptor-bound protein GRB-2, and T-cell-specific protein-tyrosine kinase ITK [13,95]. Additionally, engineering the CD3 RK motif into a proven chimeric antigen receptor derived from 41BB substantially bolstered its capability to combat tumors [96]. Whether this is related to p56^lck^ or involves a well-established role of RK motifs in endosomal/lysosomal trafficking signals, ubiquitination or clathrin-mediated endocytosis remains unclear. In this context, in NK cells, CD28ζ CARs’ recruitment of kinases, such as p56^lck^ and ZAP-70, has been reported to promote stronger CAR and ZAP-70 phosphorylation (Figure 3).

Conversely, another study indicated that CD28–CAR T-cells can operate via the Fyn kinase in the absence of p56^lck^. While p56^lck^ and p59^fyn^ have previously been shown to phosphorylate ITAMs, p59^fyn^ has also been found to negatively affect T-cell activation [97]. In knockout models, the absence of Fyn facilitates more rapid activation, increased IL-2 production, and enhanced cell division [98]. This CAR function was mediated via p59^fyn^ and requires the CD28 intracellular domain. The ability of p59^fyn^ to substitute for p56^lck^ is consistent with our previous finding that both kinases can phosphorylate the CD28 cytoplasmic tail [95]. In their model, p56^lck^-deficient CAR T-cells also exhibit robust CAR-mediated signaling with improved in vivo efficacy, reduced exhaustion and enhanced CAR memory and proliferation. In their model, p56^lck^ signaling was found to be more robust, leading to greater exhaustion. The less potent p59^fyn^ signaling promoted proliferation with less exhaustion [98]. In this case, less robust signaling led to a better outcome for CAR therapy. Whether this applies to different tumor types and in different tumor microenvironments (TEMs) is unclear. One could hypothesize that p56^lck^ signaling operates best in more restrictive TEMs where CARs struggle to become activated or to mediate effector functions. These observations offer potential opportunities for optimizing CAR design and therapeutic efficacy. Overall, the availability of src kinases such as p56^lck^ is important for the function of endogenous antigen and co-receptors in CAR signaling [99].

## 7. Conclusions

The T-cell receptor signaling pathway is an essential step in initiating cellular changes for T-cell immunity. The discovery by our lab of the CD4/CD8–p56^lck^ complexes provided the first indication of how the initiation of T-cell activation occurs and how this involves a protein-tyrosine kinase initiate phosphorylation cascade in T-cells. This finding provided a mechanism by which reporters lacking intrinsic protein-tyrosine kinase domains could elicit signal in cells and set a precedent for the discovery of other receptor-kinase interactions in other cells, such as the B-cell receptor with the Lyn kinase. The discovery of the CD4/CD8–p56^lck^ complexes also led to the identification of protein-tyrosine kinases such as ZAP-70 and ITK and downstream substrates such as phospholipase-C gamma and adaptor proteins such as LAT, SKAP1 and ADAP. The targets of CD4/CD8–p56^lck^ complexes such as TCR ITAMs and CD28 pro-survival motifs also allowed for the development of chimeric antigen receptors (CARs) and are key to immune checkpoint blockade by anti-PD-1. PD-1 interacts with phosphatases that may act to counter phosphorylation events mediated by p56^lck^ and downstream kinases. Recent advances have provided a mechanism to implicate p56^lck^ in CD28 co-receptor signaling. The relative involvement of the receptor-kinase complexes versus free p56^lck^ in the activation of T-cells in T-cell subsets and in the overall design of novel CARs in immunotherapy will be determined in future studies.

## Data Availability

Not applicable.
